# Wavelet analysis of oximetry recordings to assist in the automated detection of moderate-to-severe pediatric sleep apnea-hypopnea syndrome

**DOI:** 10.1371/journal.pone.0208502

**Published:** 2018-12-07

**Authors:** Fernando Vaquerizo-Villar, Daniel Álvarez, Leila Kheirandish-Gozal, Gonzalo C. Gutiérrez-Tobal, Verónica Barroso-García, Andrea Crespo, Félix del Campo, David Gozal, Roberto Hornero

**Affiliations:** 1 Biomedical Engineering Group, Universidad de Valladolid, Valladolid, Spain; 2 Pneumology Service, Hospital Universitario Río Hortega, Valladolid, Spain; 3 Department of Child Health, The University of Missouri School of Medicine, Columbia, Missouri, United States of America; 4 IMUVA, Instituto de Investigación en Matemáticas, Universidad de Valladolid, Valladolid, Spain; University of the Basque Country, SPAIN

## Abstract

**Background:**

The gold standard for pediatric sleep apnea hypopnea syndrome (SAHS) is overnight polysomnography, which has several limitations. Thus, simplified diagnosis techniques become necessary.

**Objective:**

The aim of this study is twofold: (*i*) to analyze the blood oxygen saturation (SpO_2_) signal from nocturnal oximetry by means of features from the wavelet transform in order to characterize pediatric SAHS; (*ii*) to evaluate the usefulness of the extracted features to assist in the detection of pediatric SAHS.

**Methods:**

981 SpO_2_ signals from children ranging 2–13 years of age were used. Discrete wavelet transform (DWT) was employed due to its suitability to deal with non-stationary signals as well as the ability to analyze the SAHS-related low frequency components of the SpO_2_ signal with high resolution. In addition, 3% oxygen desaturation index (ODI3), statistical moments and power spectral density (PSD) features were computed. Fast correlation-based filter was applied to select a feature subset. This subset fed three classifiers (logistic regression, support vector machines (SVM), and multilayer perceptron) trained to determine the presence of moderate-to-severe pediatric SAHS (apnea-hypopnea index cutoff ≥ 5 events per hour).

**Results:**

The wavelet entropy and features computed in the *D*_9_ detail level of the DWT reached significant differences associated with the presence of SAHS. All the proposed classifiers fed with a selected feature subset composed of ODI3, statistical moments, PSD, and DWT features outperformed every single feature. SVM reached the highest performance. It achieved 84.0% accuracy (71.9% sensitivity, 91.1% specificity), outperforming state-of-the-art studies in the detection of moderate-to-severe SAHS using the SpO_2_ signal alone.

**Conclusion:**

Wavelet analysis could be a reliable tool to analyze the oximetry signal in order to assist in the automated detection of moderate-to-severe pediatric SAHS. Hence, pediatric subjects suffering from moderate-to-severe SAHS could benefit from an accurate simplified screening test only using the SpO_2_ signal.

## Introduction

The American Academy of Pediatrics (AAP) defines pediatric sleep apnea-hypopnea syndrome (SAHS) as a breathing disorder characterized by recurrent episodes of complete cessation (apnea) and/or significant reduction (hypopnea) of airflow during sleep [1]. SAHS is a highly prevalent condition among children (in the range of 1% to 5%) that may lead to many adverse consequences on the overall health and quality of life, such as cognitive deficits, behavioral abnormalities, sleepiness, systemic inflammation, and cardiac and metabolic derangements [1].

The gold standard technique for pediatric SAHS diagnosis is overnight polysomnography (PSG). It involves recording a wide range of biomedical signals in a specialized sleep laboratory [2,3]. These recordings are used to score apneas and hypopneas in order to compute the apnea-hypopnea index (AHI), defined as the number of apneas and hypopneas per hour (e/h) of sleep. AHI is the clinical variable used to establish SAHS. The diagnosis of moderate-to-severe pediatric SAHS is confirmed when they present an AHI ≥5 e/h, irrespective of other co-morbidities [1]. These children are at increased risk of suffering from the major negative consequences of the disease [3–5]. Thus, to expedite the diagnosis and treatment is essential in these patients. In this sense, surgical treatment with adenotonsillectomy is consistently recommended for children suffering from SAHS with an AHI ≥5 e/h [6]. This treatment leads to an improvement in the condition in the majority of pediatric patients who suffer from moderate-to-severe childhood SAHS [1]. However, in spite of the PSG serving as the current recommended diagnostic gold standard, it is costly and complex due to the necessary equipment and trained staff, as well as highly intrusive due to the use of multiple sensors. In addition, it is a time-demanding method that shows limited availability and absent scalability, thereby delaying the diagnosis and treatment of SAHS patients [7,8].

These drawbacks have led to extensive exploration of the use of simplified diagnostic techniques [9,10]. One common approach is the analysis of a reduced set of cardiorespiratory signals involved in PSG. In this regard, overnight oximetry is a common alternative due to its reliability, simplicity, and suitability for children [7,11]. Nocturnal oximetry records the blood oxygen saturation (SpO_2_) signal, which provides a numerical measure of the oxygen content in hemoglobin [12]. Apneic events result in decreases in blood oxygen levels and such events are termed oxyhemoglobin desaturations [12]. Hence, the SpO_2_ signal contains useful information to detect pediatric SAHS. Previous studies have shown the usefulness of automated analysis of the SpO_2_ signal from nocturnal oximetry to assist in the screening of moderate-to-severe pediatric SAHS [13–19]. However, the results obtained in these studies indicate that an accurate diagnosis of pediatric SAHS is difficult, and in fact, substantially more difficult than in adults, particularly because the frequency of apneic events and reductions in SpO_2_ is markedly lower in children. Thus, further scientific evidence is still necessary before the diagnostic ability of the SpO_2_ signal can be widely implemented as a pragmatic tool to assist in an automated detection of childhood SAHS.

Different signal processing techniques have already been applied to characterize the changes produced in the SpO_2_ signal as elicited by apneic events. Conventional oximetry indices, statistical measures, nonlinear parameters, and spectral analysis from the SpO_2_ recordings have all been evaluated [13–19]. Among these approaches, the use of spectral analysis is a common choice due to the recurrence of apneic events. In this sense, previous studies have assessed features extracted from the power spectral density (PSD) and bispectrum [17–19]. However, these methods are based on the Short-Time Fourier Transform (STFT), thus having a fixed time-frequency resolution [20]. In contrast, wavelet transform (WT) offers high frequency resolution at low frequencies as well as high time resolution at high frequencies [20,21]. This property makes WT a potentially more suitable technique to accurately detect low frequency components, such as those associated with the duration of SpO_2_ desaturations. Additionally, WT is also suitable to analyze non-stationarities like those occurring in the SpO_2_ signal by apnea-hypopnea events. In this sense, wavelet analysis has proven its usefulness to detect changes produced in biomedical signals by apneic events among adult SAHS patients [22–27]. Nevertheless, only two single preliminary studies by our group evaluated the usefulness of the wavelet analysis in the detection of pediatric SAHS using the SpO_2_ signal [28,29]. Therefore, additional research is clearly needed to further corroborate previous findings in a small cohort and to assess the usefulness of wavelet analysis of SpO_2_ in the diagnosis of pediatric SAHS. Thus, we propose to develop a more exhaustive wavelet analysis with a larger database of 981 overnight SpO_2_ recordings.

We hypothesized that the multiresolution analysis afforded by the WT could provide a set of useful features to precisely characterize changes occurring in the SpO_2_ signal associated with pediatric SAHS. Consequently, the aim of this study was twofold: (*i*) to analyze oximetry dynamics by means of WT-derived features in order to characterize differences associated with the presence of SAHS; and (*ii*) to assess the usefulness of these features to assist in an automated detection of moderate-to-severe pediatric SAHS.

## Materials and methods

### Subjects and signals under study

The database is composed of 981 pediatric subjects (602 males and 379 females) ranging from 2 to 10 years of age. All children were referred to the Pediatric Sleep Unit at the University of Chicago Medicine-Comer Children’s Hospital (Chicago, IL, USA) in the context of clinical suspicion of SAHS. All legal caretakers of the children gave their informed consent as a prerequisite to be part of the study and the Ethics Committee of the hospital approved the protocols (#11-0268-AM017, # 09-115-B-AM031, and # IRB14-1241).

Children’s sleep was monitored using a digital polysomnography system (Nihon Kohden America Inc., CA, USA). SpO_2_ recordings were acquired during overnight polysomnography at sampling rates of 25, 200, or 500 Hz. In a preprocessing stage, artifacts were removed by discarding those SpO_2_ values below 50% and those intervals with a slope higher than 4%/s [30]. Then, SpO_2_ recordings were resampled to a common rate of 25 Hz, as recommended by the American Academy of Sleep Medicine (AASM) [12], and were rounded to the second decimal place in order to have the same resolution [31]. The guidelines of the AASM were used by a certified pediatric sleep specialist to quantify sleep and cardiorespiratory events. The AHI was subsequently derived in order to diagnose pediatric SAHS. An AHI of 5 e/h was the threshold used to establish moderate-to-severe SAHS because of the enhanced risk of morbidity and thus the importance of an early detection and treatment in these cases. According to this AHI-based cutoff, 405 children were in the group AHI ≥5 e/h, whereas 576 children were in the group AHI <5 e/h.

The dataset was randomly divided into an optimization set (60%) and a cross-validation set (40%) [19]. Table 1 shows demographic and clinical data of the population under study (median [interquartile range] or n (%)). No statistically significant differences (*p*-value < .01) emerged in either age or body mass index (BMI) between optimization and cross-validation groups.

**Table 1:** Demographic and clinical characteristics of the patient groups under study.

	All	Optimization set	Cross-validation set
**Subjects (n)**	981	589	392
**Age (years)**	6 [3–9]	6 [3–8]	6 [3–9]
**Males (n)**	602 (61.4%)	347 (58.9%)	255 (65.1%)
**BMI (kg/m**^**2**^**)**	17.9 [15.8–21.9]	17.6 [15.9–22.0]	18.1 [18.1–21.7]
**AHI (e/h)**	3.8 [1.5–9.3]	4.1 [1.7–9.9]	3.3 [1.4–7.8]
**Group AHI <5 e/h (n)**	576 (58.7%)	330 (56.0%)	246 (62.8%)
**Group AHI ≥5 e/h (n)**	405 (41.3%)	259 (44.0%)	146 (37.2%)

BMI: Body Mass Index; AHI: Apnea Hypopnea Index. Data are presented as median [interquartile range] or n (%)

### Methods

Our methodology is divided into three steps: feature extraction, selection, and classification. In the first step, the wavelet transform was applied to analyze each SpO_2_ signal. A set of features was computed using the discrete wavelet transform (DWT) to characterize the changes produced in SpO_2_ recordings due to SAHS. In addition, 3% oxygen desaturation index (*ODI*3), statistical moments in the time domain and PSD features, which are common features from the SpO_2_ signal [17,19], were obtained to compose a wide initial feature set with relevant as well as complementary information. In the second step, a feature subset was selected using the fast correlation-based filter (FCBF) method [32]. Finally, binary logistic regression (LR) [33], support vector machines (SVM) [34] and multi-layer perceptron (MLP) neural network [35] classifiers were trained using this selected feature subset in order to detect moderate-to-severe pediatric SAHS.

**Fig 1:**
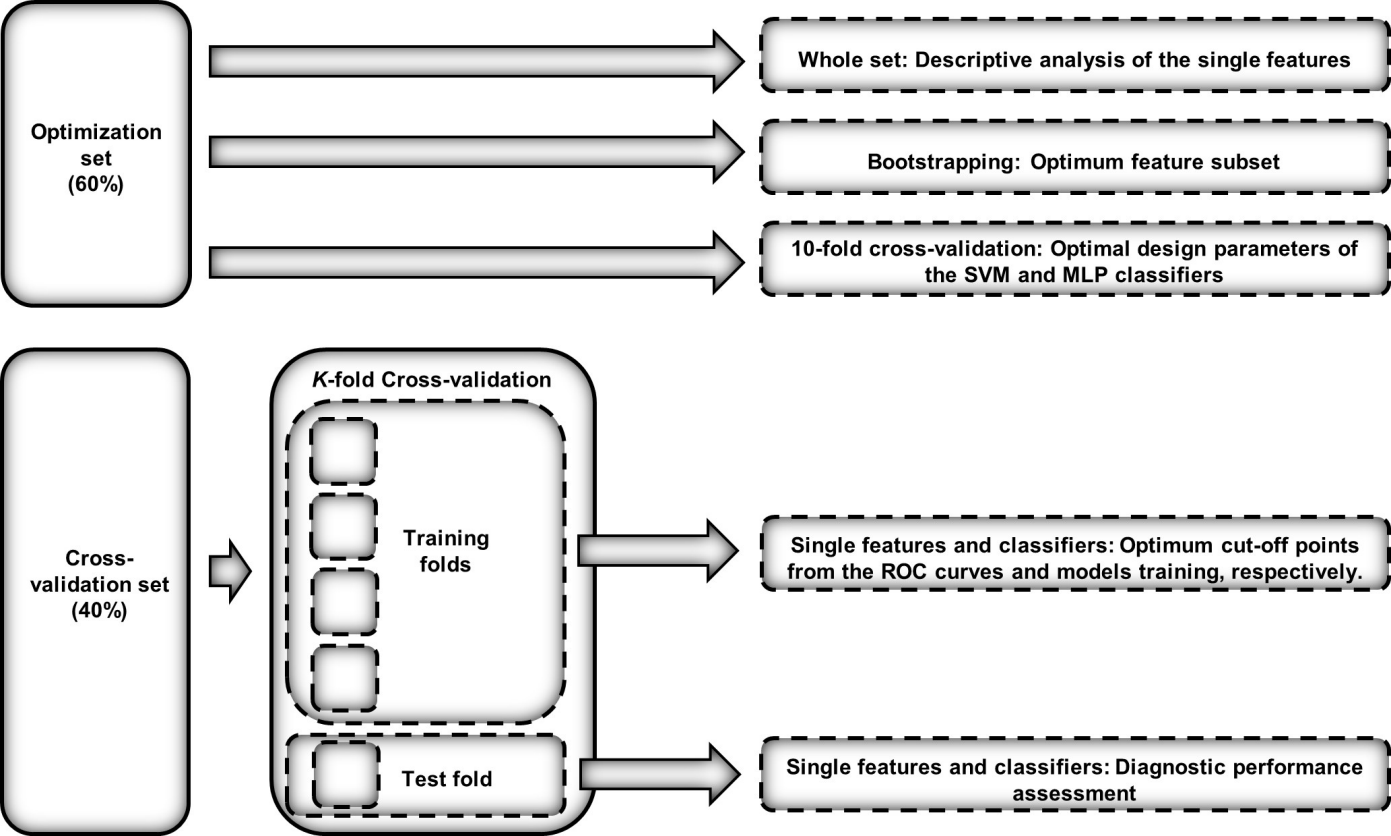
Validation approach employed in each methodological step of the study.

Fig 1 shows the validation approach employed in each methodological step. The first set (optimization set) was employed to perform descriptive analysis of the extracted features, select a subset of features with FCBF, and select the optimal design parameters of the SVM and MLP classifiers. Bootstrapping has been employed in the feature selection stage, in order to avoid overfitting [36]. In the same way, 10-fold stratified cross validation has been applied to optimize the design parameters of SVM and MLP. The second set (cross-validation set) was used to evaluate the diagnostic performance of the single features and classifiers. Stratified *K*-fold cross validation (*K* = 5) was applied for this purpose [37].

#### Feature extraction

**Discrete Wavelet Transform**

WT can be seen as the decomposition of a signal *x*(*t*) onto a set of basis functions, called wavelets [20]. Wavelets are obtained by time translations and scaling of a unique function called the mother wavelet. WT can be seen as an extension of the Fourier transform where, instead of analyzing a single scale, a multiscale analysis is performed. This multiscale property of the WT allows decomposing a signal into a set of scales, where each scale analyzes a different frequency range of the signal. WT can be continuous (Continuous Wavelet Transform, CWT) or discrete (DWT), depending on the scale and translation values [20]. CWT computes WT for each scale, whereas DWT only computes WT for dyadic (power of 2) scales, thus presenting lower complexity and higher computational efficiency than CWT [38]. Consequently, DWT was chosen in this study. In addition, it has previously shown its usefulness to detect different frequency components in physiological signals associated to SAHS events in adult patients [22–27].

**Fig 2:**
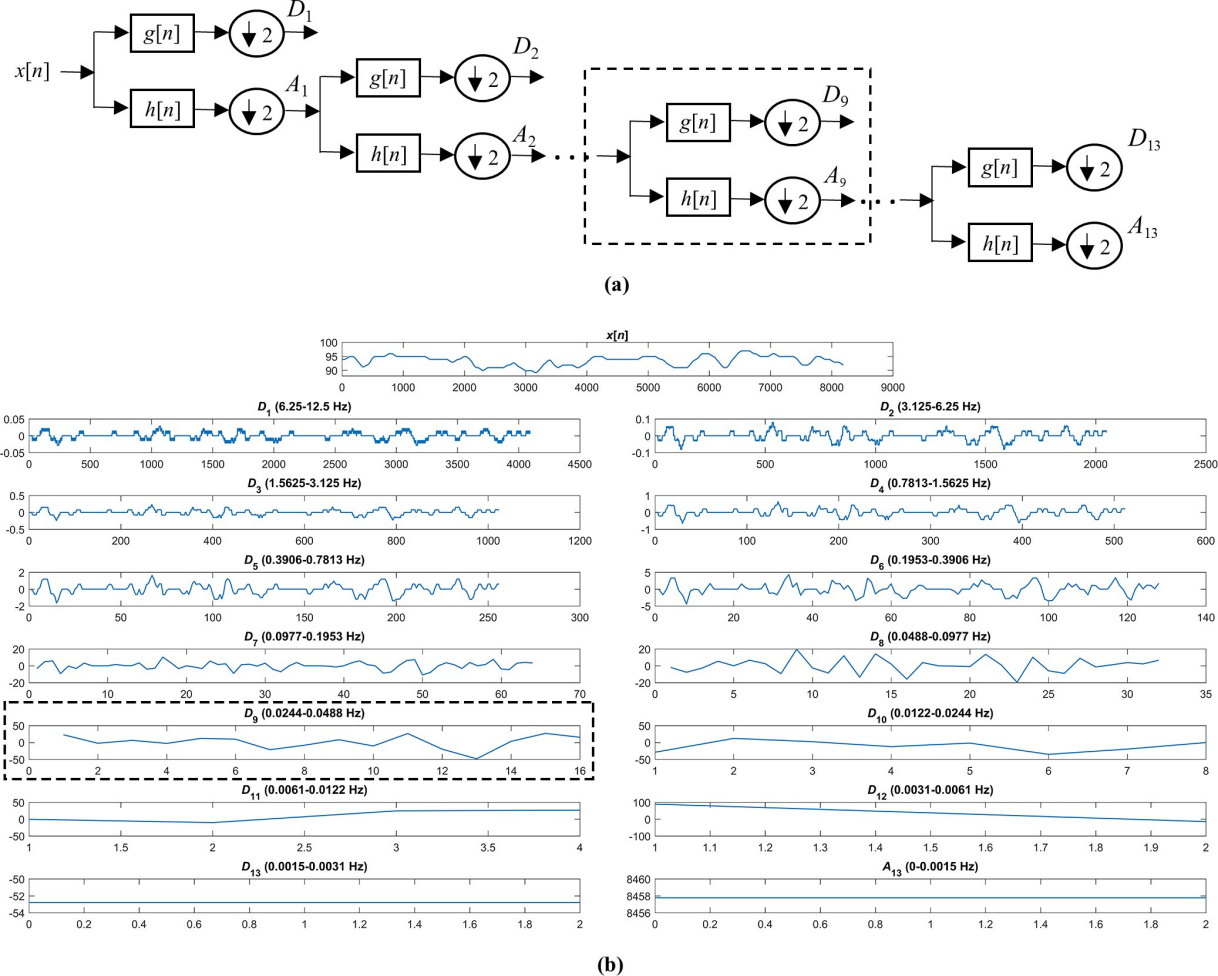
DWT computation. (A) Decomposition process of a signal using DWT. (B) Original SpO_2_ signal, detail signals at each decomposition level and approximation signal at the maximum level of the decomposition.

Fig 2 shows how DWT is computed. In Fig 2A, the decomposition process of a SpO_2_ signal *x*[*n*] using DWT, the so-called subband coding scheme, is illustrated. It is a filter-bank tree where each stage consists of a high pass-filter *g*[*n*] (the mother wavelet) and a low pass filter *h*[*n*] (the mirror version of the mother wavelet), followed by a subsampling process of factor two [20]. The relationship between these two filters is as follows [20]:

$$g[L-1-n]={\left(-1\right)}^{n}\cdot h[n],$$ (1)

where *L*, an even number, is the length of the filter. First, *x*[*n*] is decomposed in an approximation signal (lowpass version), *A*_1_, and a detail signal (highpass version), *D*_1_. Then, *A*_1_ is further decomposed into another approximation signal, *A*_2_, and another detail signal, *D*_2_. Each iteration increases the frequency resolution of the approximation and the detail version by two, as well as decreases the number of samples of both approximation and detail signals. This process continues until the maximum detail level of the signal, *N* = *log*2(*M*) is reached, being *M* the length of *x*[*n*] [39]. At each level (*i* = 1, 2, …, *N*), the approximation signal, *A*_*i*_, and the detail signal, *D*_*i*_, can be computed as follows:

$$D_{i}[k]=\sum_{n}A_{i-1}[n]\cdot g[2k-n],$$ (2)

$$A_{i}[k]=\sum_{n}A_{i-1}[n]\cdot h[2k-n].$$ (3)

where *A*_*i*-1_ is the approximation signal in the level *i*-1. In the level 1, *A*_0_ is the original signal *x*[*n*]. Fig 2B shows an example of SpO_2_ signal, *x*[*n*], the detail signal *D*_*i*_ obtained at each level *i* of the DWT decomposition, and the approximation signal *A*_*N*_ obtained at the level *N* of the DWT decomposition.

DWT was applied to the upper power of 2 for 5 minute segments (*M* = 2^13^ samples (5.5 minutes)) and, consequently, *N* = 13 [23]. In this study, the Haar wavelet was chosen as mother wavelet. The reason is twofold [27]: (*i*) its suitability for picking up abrupt changes, which is appropriate to detect the changes produced in the SpO_2_ values due to apneic events; and (*ii*) its smoothing feature, which does not distort the original form of the SpO_2_ signal. At each level of the decomposition, detail coefficients contain information about a different frequency band, as stated in Fig 2B. We focused on the detail coefficients of the level 9 (*D*_9_, i.e., 0.0244–0.0488 Hz), since it is the level which is contained in the band of interest previously related to the recurrence of apneic events [18]. SpO_2_ signal presents both drops and rises associated to apneic events, which result in decreased and increased values in *D*_9_ coefficients, respectively. The information contained in the *D*_9_ coefficients may be canceled due to the presence of both positive and negative values, such as mean or skewness. To avoid this, the absolute values of the DWT coefficients were used. The following seven features were extracted from the DWT coefficients:

- Statistical moments of the *D*_9_ coefficients (Mean (*M*1_*D*9_), variance (*M*2_*D*9_), skewness (*M*3_*D*9_) and kurtosis (*M*4_*D*9_)). *M*1_*D*9_–*M*4_*D*9_ measure the central tendency, dispersion, asymmetry and peakedness of the data, respectively.
- Maximum amplitude of the *D*_9_ coefficients (*Max*_*D*9_). It quantifies the highest amplitude in this frequency band.
- Energy of the *D*_9_ coefficients (*En*_*D*9_). It measures the averaged quadratic amplitude of the signal in *D*_9._ It is computed as follows:

$$En_{D_{9}}=\sum_{k}{\left|D_{9}[k]\right|}^{2},$$ (4)

- Wavelet Entropy (*WE*), which measures the irregularity introduced in the DWT. It was extracted in order to obtain information about the changes produced in the energy distribution of the different detail levels of the DWT of the SpO_2_ signal by apneic events [39]. It is computed as follows:

$$WE=-\sum_{i=1}^{N}p_{i}\cdot \ln\left(p_{i}\right),$$ (5)

where *p*_*i*_ is the relative wavelet energy at the detail level *D*_*i*_:

$$p_{i}=\frac{En_{D_{i}}}{\sum_{i=1}^{N}En_{D_{i}}},$$ (6)

Where *En*_*Di*_ is the wavelet energy at the detail level *D*_*i*_:

$$En_{D_{i}}=\sum_{k}{\left|D_{i}[k]\right|}^{2},$$ (7)

**Conventional features from the SpO_2_ signal**

In order to enhance the diagnostic ability of our proposal, the following features, that are common parameters of the oximetry signal [17,19], were computed:

- *ODI*3. It was estimated as the number of desaturations of at least 3% from preceding baseline per hour of recording [40]. This parameter has shown its usefulness in clinical studies, even though it underestimates AHI [13–15].
- Statistical moments. First-to-fourth order statistical moments were computed from the SpO_2_ signal in the time domain (*M*1_*T*_ -*M*4_*T*_): mean (*M*1_*T*_), variance (*M*2_*T*_), skewness (*M*3_*T*_), and kurtosis (*M*4_*T*_) [17,19]. These features measure the central tendency, dispersion, asymmetry, and peakedness of the data, respectively.
- PSD features. PSD was estimated using the Welch’s method (2^13^-sample Hamming window, 50% overlap and 2^14^-points DFT) [41]. The following features were obtained: first-to-fourth order statistical moments (*M*1_*PSD*_- *M*4_*PSD*_) and maximum amplitude (*Max*_*PSD*_) from the band of interest determined in [18] (0.018–0.050 Hz) and spectral entropy (*SE*_*PSD*_) in the full spectrum. These features provide information about the recurrence and duration of apneic events.

#### Feature selection: Fast Correlation-Based Filter (FCBF)

The FCBF method was applied to select a subset of relevant and non-redundant features [32]. FCBF is a feature selection algorithm that has previously shown its usefulness in the context of pediatric SAHS [18,19]. First, FCBF computes the symmetrical uncertainty (*SU*) between each feature (*x*_*i*_) and the AHI (*y*). *SU* is a normalization of the information gain between two variables. *SU* is computed as follows [32]:

$$SU(x_{i},y)=2\left(\frac{IG(x_{i}|y)}{H(x_{i})+H(y)}\right),i=1,2,...,N,$$ (8)

where *IG*(*x*_*i*_|*y*) = *H*(*x*_*i*_)—*H*(*x*_*i*_|*y*), *N* is the total number of features extracted and *H* refers to Shannon’s entropy [32]. According to their *SU* value (between 0 and 1), features are ranked from the most relevant (highest *SU* with the AHI) to the least relevant one (lowest *SU* with the AHI). Then, a redundancy analysis is performed. *SU* between each pair of features (*x*_*j*_, *x*_*i*_) is computed. Features *x*_*j*_ sharing more information with a more relevant one than with the AHI (*SU* (*x*_*j*_| *x*_*i*_) ≥ *SU*(*x*_*j*_|*y*)) were discarded. Finally, an optimum subset composed of the features not discarded in this process is obtained.

A bootstrap approach was employed in order to obtain a subset of features independent of a particular dataset. In this regard, FCBF was applied to 1000 bootstrap replicates built with a sample with replacement procedure from the optimization set [42,43]. Those variables that were selected with FCBF more than 500 times (50% of runs) formed the feature subset [18,19].

#### Feature classification

In this study, we employed LR, SVM, and MLP, which are well-known algorithms in the context of binary classification. Particularly, these algorithms were applied to assign each subject to the groups AHI <5 e/h and AHI ≥5 e/h [33–35].

**Logistic regression**

LR is a standard machine learning approach for binary classification. Given a set of input features, LR estimates the *posterior* probability of a given instance (subject) belonging to one of two mutually exclusive groups (AHI <5 e/h and AHI ≥5 e/h) by the use of the logistic function [33]:

$$p(C_{l}|x_{k})=\frac{1}{1+e^{-(\beta_{0}+\beta_{1}x_{1,k}+...+\beta_{N}x_{N,k})}},$$ (9)

where *C*_*l*_ represents the two groups (AHI <5 e/h and AHI ≥5 e/h), *β* = *β*_0_, *β*_1_, …, *β*_*N*_ are the coefficients of the model for each input feature, *x*_*k*_ = *x*_1,*k*_, …, *x*_*N*,*k*_, is the input pattern for the instance *k*, and *N* is the number of features. A Bernoulli distribution is used to model the probability density function and *β* coefficients are optimized using the maximum likelihood ratio [33].

**Support vector machines**

A SVM is a binary classifier that searches for the best hyperplane that separates instances from the classes under study [34]. The hyperplane has the following expression [34]:

$$y(x,w)=w^{T}ɸ(x)+w_{0},$$ (10)

where *x* ϵ ℝ^N^ is the input pattern of dimension *N* (number of features), *ɸ*(*x*) ϵ ℝ^P^ transforms the data into a high-dimensional space *P*>*N*, and *w* is the weight vector. The weight vector *w* is optimized in order to maximize the margin of separation between the two groups [34]. A regularization parameter *C* was applied to control the trade-off between maximizing the margin of separation between groups and obtaining a good generalization ability in an independent set [34]. The optimization problem of SVM is formulated using Lagrange multipliers:

$$y(x,w)=\sum_{i\in S}\eta^{i}t^{i}K(x^{i},x)+w_{0},$$ (11)

where *S* is a subset of the indices {1, …, *L*} corresponding to the non-zero Lagrange multipliers (support vectors) *η*^*i*^, *L* is the number of observations in the training set, *t*^*i*^ are the output labels (±1 for the AHI ≥5 and AHI <5 e/h groups), and *K*(∙,∙) is the kernel function in the transformed space. In this study, a linear kernel was used, which has previously shown its usefulness in the context of adult SAHS [44]. The value of *C* was optimized by means of 10-fold cross-validation using the optimization set.

**Multi-layer perceptron neural network**

A MLP is an artificial neural network arranged in several fully connected layers: input, hidden, and output layers [35]. These layers are composed of computing units called perceptrons or neurons. Each neuron consists of an activation function *g*^*k*^{.} and adaptive weights *w*_*kj*_ that interconnect the neuron with neurons from the subsequent layer [35]. The input layer was composed of one neuron for each input feature. Additionally, a configuration with one single hidden layer with a hyperbolic tangent activation function was applied since it provides a fast convergence for the training algorithm [35]. This configuration can provide universal approximation to any continuous function with the only condition that there are enough hidden units [35,45]. Finally, two neurons composed the output layer, since our problem is a binary classification task. A logistic sigmoid activation function has been used in the output layer, because it allows the output neurons to be interpreted probabilistically [35]:

$$y_{k}=g^{k}\left\{\sum_{j=1}^{N_{H}}w_{kj}g^{j}\{\sum_{i=1}^{N}w_{ji}x_{i}+b_{j}\}+b_{k}\right\},$$ (12)

where y_k_ are the outputs neurons, *w*_*kj*_ are the weights connecting the hidden layer to the output layer, *w*_*ji*_ are the weights connecting the input layer to the hidden layer, *b*_*j*_ and *b*_*k*_ are the bias associated to the hidden and the output units, respectively, *x*_*i*_ is the feature *i*, *g*^*k*^{.} and *g*^*j*^{.} are the activation functions of the output and hidden layer, respectively, *N*_*H*_ is the number of neurons in the hidden layer, and *N* is the number of input features [35]. Random initialization was performed for the weights of the network. Then, the scaled conjugate gradient algorithm with weight-decay regularization was used to optimize the weights [35]. *N*_*H*_ and the regularization parameter (*α*) were optimized by means of 10-fold cross-validation using the optimization set.

#### Statistical analysis

The software tools Matlab version R2017a was used for performing signal processing and statistical analyses. Normality and homoscedasticity tests showed that extracted parameters were not normality distributed and had different variances. Consequently, the Mann-Whitney *U* test was applied to search for statistical significant differences in the extracted features (*p*-value <0.01) between groups. Diagnostic performance was assessed by means of sensitivity (Se, percentage of patients with an AHI ≥5 e/h correctly classified), specificity (Sp, percentage of children with an AHI <5 e/h correctly classified), positive predictive value (PPV, proportion of subjects classified as positive that are true positives), negative predictive value (NPV, proportion of subjects classified as negative that are true negatives), positive likelihood ratio (LR+, likelihood ratio for subjects classified as positive), negative likelihood ratio (LR-, likelihood ratio for subjects classified as negative), and accuracy (Acc, percentage of subjects correctly classified).

*K*-fold stratified cross validation (*K* = 5) was applied to assess the performance of the extracted features and the binary classifiers [37]. The cross-validation set was randomly divided into *K* subsets, preserving the proportion of subjects belonging to the groups AHI <5 e/h and AHI ≥5 e/h. *K*-1 folds formed the training folds (80% of the cross-validation set), whereas the remaining one formed the test fold (20% of the cross-validation set). Accordingly, Receiver Operating Characteristics (ROC) curves were used to obtain optimum classification cut-off points for the single features using the *K*-1 training folds. Similarly, the classification algorithms were trained using the training folds. Then, the diagnostic performance of the single features and the LR, SVM, and MLP classifiers was measured using the test fold. This process was repeated *K* times, so each fold was considered once as the test fold. Finally, all the metrics are averaged across the *K* = 5 iterations.

## Results

### Feature separability

**Fig 3:**
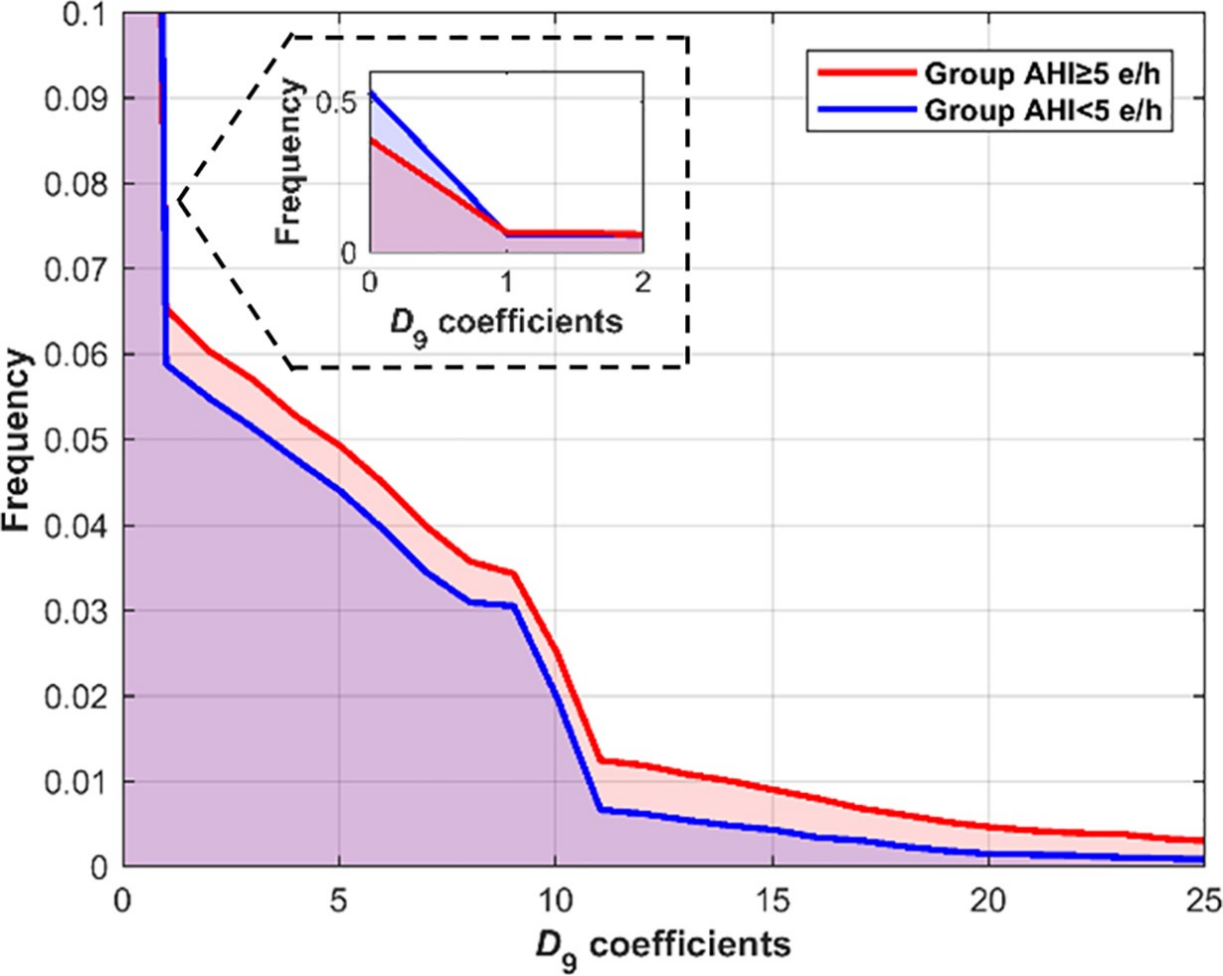
Histogram of the *D*_9_ coefficients for each group in the optimization set.

A total of seven DWT-derived features were obtained for each SpO_2_ recording (S1 Table). Fig 3 shows the histogram of the *D*_9_ coefficients in the optimization set for the groups AHI <5 e/h and AHI ≥5 e/h. It can be observed that *D*_9_ coefficients are more concentrated near zero in the AHI <5 e/h group, whereas in the group AHI ≥5 e/h these coefficients are more disperse. Table 2 shows the median and interquartile range of all these extracted features in the optimization set for both groups. All features showed significant statistical differences (*p*-value <0.01) between groups. *M*1_*D*9_, *M*2_*D*9_, *Max*_*D*9_, *En*_*D*9_, and *WE* showed higher values in the AHI ≥5 e/h group, whereas *M*3_*D*9_ and *M*4_*D*9_ showed higher values in the AHI <5 e/h group. ODI3, statistical moments and PSD features were also computed for each SpO_2_ recording (S1 Table).*ODI*3, 3 out of 4 statistical moments (*M*1_*T*_, *M*2_*T*_, and *M*3_*T*_) and 3 out of 6 spectral features (*M*1_*PSD*_, *M*2_*PSD*_, and *Max*_*PSD*_) also showed significant statistical differences (*p*-value <0.01), which agrees with previous studies [17,18].

**Table 2:** DWT-derived features for each group in the optimization set.

Feature	Group AHI <5 e/h	Group AHI ≥5 e/h	*p*-value
***M1***_***D9***_	3.04 [2.26 3.92]	5.36 [3.77 7.70]	*p* << .01
***M2***_***D9***_	3.78 [3.23 4.63]	5.73 [4.30 7.57]	*p* << .01
***M3***_***D9***_	1.31 [1.20 1.44]	1.19 [1.06 1.32]	*p* << .01
***M4***_***D9***_ **(10**^**2**^**)**	3.58 [1.03 7.69]	0.06 [0.04 2.69]	*p* << .01
***Max***_***D9***_ **(10**^**1**^**)**	1.23 [1.04 1.55]	1.96 [1.42 2.62]	*p* << .01
***En***_***D9***_ **(10**^**3**^**)**	0.54 [0.37 0.89]	1.54 [0.78 2.96]	*p* << .01
***WE* (10**^**−4**^**)**	1.83 [1.18 2.86]	4.27 [2.52 9.41]	*p* << .01

### Optimum feature subset

FCBF was applied to each bootstrap replicate from the optimization set, each one composed of all the extracted features (*ODI*3, statistical moments, PSD, and DWT features). *ODI*3, 1 statistical moment (*M*2_*T*_), 3 features from PSD (*M*2_*PSD*_, *M*3_*PSD*_, and *Max*_*PSD*_), and 3 DWT-derived features (*M*3_*D*9_, *En*_*D*9_ and *WE*) were selected more than 50% of times (500) (S2 Table). Thus, these features formed the selected feature subset [18,19]. Notice that features from all the different methodological approaches were selected.

### Classification models optimization

LR, SVM, and MLP classifiers were designed using the selected feature subset obtained with FCBF (*ODI*3, *M*2_*T*_, *M*2_*PSD*_, *M*3_*PSD*_, and *Max*_*PSD*_, *M*3_*D*9_, *En*_*D*9_, and *WE*). Optimum values for the design parameters of the SVM (regularization parameter: *C*) and MLP classifiers (number of neurons in the hidden layer: *N*_*H*_; regularization parameter: *α*) were obtained as those for which the Acc of the classifiers was the highest in the optimization set. Concerning SVM, the following values of *C* were assessed: 10^−5^, 10^−4^, 10^−3^, …, 10^4^, 10^5^. The optimum value of the input parameter *C* was 10^3^, which maximizes Acc. Regarding MLP, *N*_*H*_ was varied from 2 up to 50 and *α* was varied from 0 up to 10. Since the network depends on the initial random values of the weights, the accuracy was computed and averaged for a total of 10 runs for each pair *N*_*H*_-*α*. Finally, user-dependent network parameters *N*_*H*_ = 5 and *α* = 1 were chosen since this pair reached the highest accuracy.

### Diagnostic performance

The value of all the extracted features (*ODI*3, statistical moments, PSD, and DWT features) and the classification score of the LR, SVM, and MLP classifiers were obtained for each subject in the cross-validation set (S3 Table). Table 3 shows the diagnostic ability of each single feature in the cross-validation set obtained using optimum cut-off point obtained from the ROC curve. Most of the DWT-derived features (5 out of 7) showed accuracies near 80%. In this regard, *Max*_*D9*_ achieved the highest performance (81.7±5.6% Acc, with 75.4±7.1% Se and 85.4±6.8% Sp), outperforming statistical moments and PSD features. Only *ODI*3 achieved slightly higher Acc than *Max*_*D9*_, reaching 81.9±7.2% Acc (78.1±7.3% Se and 84.2±8.1% Sp). Table 4 shows the diagnostic performance of LR, SVM, and MLP classifiers, which were trained using the selected feature subset (*ODI*3, *M*2_*T*_, *M*2_*PSD*_, *M*3_*PSD*_, and *Max*_*PSD*_, *M*3_*D*9_, *En*_*D*9_, and *WE*) obtained with FCBF, in the cross validation set. These classifiers showed high diagnostic performance, outperforming all the extracted features in terms of Sp, PPV, LR+, and Acc. SVM achieved the highest accuracy (84.0±5.2% Acc, with 71.9±4.4% Se and 91.1±7.2% Sp) for the cutoff of 5 e/h.

**Table 3:** Diagnostic ability of the proposed features (*ODI*3, statistical moments, PSD, and DWT) in the cross-validation set.

Feature	Se (%)	Sp (%)	PPV (%)	NPV (%)	LR+	LR-	Acc (%)
***ODI*3**	78.1±7.3	84.2±8.1	75.2±10.2	86.5±5.0	6.1±2.9	0.27±0.11	81.9±7.2
***M*1**_***T***_	62.3±6.8	65.0±2.6	51.4±2.1	74.6±3.6	1.8±0.2	0.58±0.10	64.0±2.3
***M*2**_***T***_	72.6±13.6	67.1±6.6	56.7±2.8	81.2±6.6	2.2±0.3	0.40±0.17	69.2±3.1
***M*3**_***T***_	65.0±8.5	61.4±6.8	50.1±2.8	74.9±2.8	1.7±0.2	0.57±0.09	62.7±2.7
***M*4**_***T***_	60.9±15.6	49.9±8.4	41.6±5.0	69.0±7.5	1.2±0.3	0.78±0.26	54.0±5.2
***M*1**_***PSD***_	75.3±7.9	82.5±7.4	73.0±8.5	85.1±3.5	5.3±3.1	0.30±0.08	79.9±3.8
***M*2**_***PSD***_	69.8±7.3	83.4±5.2	71.8±6.2	82.5±3.0	4.5±1.4	0.36±0.08	78.3±3.2
***M*3**_***PSD***_	47.2±11.7	58.1±11.9	40.4±4.1	65.0±2.8	1.2±0.2	0.91±0.12	54.1±4.5
***M*4**_***PSD***_	63.6±8.3	47.1±6.2	41.7±4.2	68.7±6.1	1.2±0.2	0.79±0.23	53.3±5.0
***Max***_***PSD***_	78.1±8.8	75.2±9.9	66.2±6.9	85.6±3.6	3.5±1.1	0.29±0.09	76.3±4.3
***SE***_***PSD***_	48.6±14.4	61.8±11.8	43.0±4.8	67.3±3.3	1.3±0.3	0.82±0.12	56.9±4.2
***M*1**_***D9***_	73.4±9.1	82.6±7.8	72.2±10.2	84.0±5.1	5.2±2.7	0.32±0.12	79.1±6.2
***M*2**_**D9**_	74.7±6.1	81.7±6.5	71.5±6.9	84.6±3.0	4.6±1.7	0.31±0.07	79.1±3.3
***M3***_**D9**_	58.3±9.2	63.4±6.5	48.7±3.1	72.1±3.3	1.6±0.2	0.66±0.10	61.5±3.2
***M*4**_**D9**_	71.2±6.7	64.6±5.7	54.6±3.3	79.2±4.0	2.0±0.3	0.45±0.10	67.1±3.5
***Max***_**D9**_	75.4±7.1	85.4±6.8	76.0±9.0	85.4±4.3	6.2±2.8	0.29±0.10	81.7±5.6
***En***_**D9**_	78.8±4.4	81.7±5.2	72.2±5.5	86.7±2.4	4.6±1.4	0.26±0.05	80.6±3.4
***WE***	76.0±8.2	78.4±5.6	68.0±3.8	84.9±3.5	3.6±0.7	0.30±0.09	77.6±2.5

**Table 4:** Diagnostic ability of the LR, SVM, and MLP models in the cross-validation set.

Feature	Se (%)	Sp (%)	PPV (%)	NPV (%)	LR+	LR-	Acc (%)
***LR***	72.6±4.7	90.2±6.2	82.3±8.8	84.7±2.8	9.8±5.5	0.31±0.06	83.7±4.9
***SVM***	71.9±4.4	91.1±7.2	83.8±10.8	84.5±2.6	14.6±12.9	0.31±0.06	84.0±5.2
***MLP***	73.3±6.6	89.0±6.9	80.7±9.2	84.9±3.3	9.0±5.8	0.30±0.08	83.2±5.2

## Discussion

In the present study, we examined the usefulness of wavelet analysis to identify features that characterize oximetry dynamics in order to expedite detection of moderate-to-severe pediatric SAHS. *WE* and features from the coefficients in *D*_9_ (*M*1_*D*9_-*M*4_*D*9_, *Max*_*D*9_, and *En*_*D*9_) were obtained from the DWT of each SpO_2_ recording. *D*_9_ (0.0244–0.0488 Hz) was chosen according to a previous study in the context of pediatric SAHS [18], and is related to the duration and frequency of the SpO_2_ desaturations associated with apneic events [40]. Statistically significant differences (*p*-value < 0.01) emerged in all DWT-derived features between the groups AHI <5 e/h and AHI ≥5 e/h in the optimization set (Table 2). The higher values showed by *M*1_*D*9_, *Max*_*D*9_, and *En*_*D*9_ in the AHI ≥5 e/h group agree with a higher amplitude of the histogram for high values of the *D*_9_ coefficients in this group. In addition, the SpO_2_ drops and rises caused by apneic events are reflected in a higher dispersion in the histogram of *D*_9_ coefficients, as reported by the higher values of *M*2_*D*9_ in the AHI ≥5 e/h group. In contrast, the lower values that *M*3_*D*9_ and *M*4_*D*9_ as reflected in the AHI ≥5 e/h group indicate that the variations produced in the SpO_2_ signal due to apneic events result in values less proximal to zero in the histogram of the *D*_9_ coefficients. Finally, the higher irregularity reported by *WE* in the SAHS positive group suggests that apnea-hypopnea events alter the energy distribution of the whole DWT profile of the SpO_2_ signal.

Regarding the diagnostic performance of the proposed features, *ODI*3 and *M*ax_*D*9_ reached similar Acc in the cross-validation set, higher than the remaining features. In addition, higher accuracies were generally obtained with the DWT-derived features with respect to statistical moments and features from PSD. This suggests that DWT is a useful approach to analyze the changes produced in the SpO_2_ signal associated to SAHS. In the feature selection stage, a feature subset composed of *ODI*3 (conventional oximetric index); *M*2_*T*_ (time); *M*2_*PSD*_, *M*3_*PSD*_, and *Max*_*PSD*_ (PSD), and *M*3_*D*9_, *En*_*D*9_ y *WE* (DWT) was obtained with FCBF. LR, SVM, and MLP models built with this subset obtained high diagnostic performance for the detection of moderate-to-severe SAHS (AHI ≥5 e/h), improving the diagnostic ability of the single features (Table 3) in terms of Sp, PPV, LR+, and Acc. It is worthy to note that the SVM model achieved the highest average Acc (84.0%), Sp (91.1%), PPV (83.8%), and LR+ (14.6) among the single features and binary classifiers. In addition, SVM reached similar NPV and LR- to LR, MLP, *ODI*3 and the remaining features. A high LR+ is especially important for screening tests [17,46]. In this sense, a LR+ greater than 10 is considered to provide strong evidence to confirm diagnoses [46]. Thus, our method is especially useful to confirm the presence of pediatric SAHS.

Three DWT features were involved in the feature subset obtained with FCBF: *M*3_*D*9_, *En*_*D*9_ and *WE*. As aforementioned, these features provide information about the concentration of the *D*_9_ coefficients near zero (*M*3_*D*9_), the amplitude of the *D*_9_ coefficients (*En*_*D*9_), and the irregularity of the distribution of the whole DWT profile of the SpO_2_ signal (*WE*). According to our results, *M*3_*D*9_, *En*_*D*9_ and *WE* provide both relevant and complementary (non-redundant) information on the changes occurring in the SpO_2_ signal due to SAHS. This is consistent with the different properties of the SpO_2_ signal these DWT-derived features quantify. The fact that a high performance was reached with the three classification algorithms reinforces the notion that DWT is a useful method to analyze the SpO_2_ signal in the context of pediatric SAHS.

To the best of our knowledge, this is the first study assessing wavelet analysis of SpO_2_ recordings in the context of pediatric SAHS. Our results suggest that DWT is an appropriate tool to analyze the low frequency components of the SpO_2_ signals related to the duration of the desaturations caused by apnea-hypopnea events since it provides high resolution at low frequencies of the power spectrum [20,21]. This assumption is further supported by previous studies, whereby DWT was also applied to quantify the frequency components of different biomedical signals associated to respiratory events in the context of adult SAHS [23,24]. Additionally, the favorable performance of our approach may be due to the suitability of the WT to analyze non stationary properties of a signal [20,21], which is appropriate to events such as the non-stationary changes of the SpO_2_ signal associated with apneic events. The high resolution afforded by WT at low frequencies, as well as its suitability to analyze non-stationary signals clearly support the contention that DWT is more appropriate than conventional spectral analysis techniques to analyze the SpO_2_ signal [20,21].

Table 5 shows the performance of previous studies focused on the automated analysis of SpO_2_ as an alternative to PSG in the screening of moderate-to-severe pediatric SAHS [13–19]. Oxygen desaturation index and clusters of desaturations have been employed for this task [13–16]. Kirk *et al*. [13] applied *ODI*3, reaching 67.0% Se, 60.0% Sp, and 64.0% Acc. Tsai *et al*. [14] obtained 83.8% Se, 86.5% Sp, and 85.1% Acc using 4% ODI (*ODI*4). However, *ODI*4 cutoff values were optimized and validated using the same population, such that no true post-hoc verification was achieved. Chang *et al*. [15] combined *ODI*3 with common symptoms to assess a discriminative score, reaching 60% Se, 86% Sp, and 72% Acc. Pia-Villa *et al*. [16] reported 69.4% Acc (40.6% Se and 97.9% Sp) combining clusters of desaturations and clinical history in a discriminative score. Our approach achieved a high the diagnostic performance while also strengthening its validity since the methods were derived using not only a much larger sample size, but also applying a cross validation approach to validate the results.

**Table 5:** Summary of the state-of-the-art studies in the context of detection of moderate-to-severe pediatric SAHS using SpO_2_ recordings.

Studies	Subjects (n)	Methods	Validation	Se (%)	Sp (%)	Acc (%)
**Kirk *et al*. [13]**	58	*ODI*3	Direct validation**	67.0	60.0	64.0*
**Tsai *et al*. [14]**	148	*ODI*4	No	83.8	86.5	85.1*
**Chang *et al*. [15]**	141	*ODI*3 and symptoms	Direct validation**	60.0	86.0	72.0*
**Pia-Villa *et al*. [16]**	268	Clusters of desaturations and clinical history	Direct validation**	40.6*	97.9*	69.4*
**Álvarez *et al*. [17]**	50	Statistical moments, spectral, nonlinear features, and classical indices	Bootstrap 0.632	82.2	83.6	82.8
**Vaquerizo-Villar *et al*. [18]**	298	Bispectrum, PSD, *ODI*3, anthropometric variables	Feature optimization- training-test	61.8	97.6	81.3
**Hornero *et al*. [19]**	4191	Statistical moments, PSD, nonlinear features, and *ODI*3	Training-test	68.2	87.2	81.7
**Our proposal**	981	*ODI*3, Statistical moments, PSD, and DWT features	Optimization- cross validation	71.9	91.1	84.0

* Computed from reported data

** Direct validation of a scoring criteria against AHI from PSG.

In order to increase the diagnostic ability of the SpO_2_ signal, conventional oximetric indices have been combined with features from other signal processing approaches in studies developed by our group [17–19]. Álvarez *et al*. [17] assessed LR models fed with conventional oximetric indices, statistical parameters, PSD, and nonlinear features. These models were validated using a bootstrap procedure, reaching 82.8% Acc (82.2% Se and 83.6% Sp). Vaquerizo-Villar *et al*. [18] assessed the usefulness of oximetry bispectrum. A multiclass multi-layer perceptron (MLP) model fed with *ODI*3, anthropometrical variables, PSD, and bispectral features reached 61.8% Se, 97.6% Sp, and 81.3% Acc in an independent test set, outperforming a MLP classifier built without bispectral features. Finally, Hornero *et al*. [19] analyzed 4,191 SpO_2_ recordings obtained from 13 sleep laboratories in a multicenter international study. A MLP regression model with *ODI*3 and the skewness of the PSD reached 68.2% Se, 87.2% Sp, and 81.7% Acc. In contrast with the findings of these studies, our current results achieved improved diagnostic ability for the screening of moderate-to-severe SAHS with the use of DWT-derived features. This suggests that wavelet analysis could enhance the detection of this clinically important and vulnerable group of SAHS severity from single-channel oximetry recordings. In these patients, it is essential to early detect this condition, since they are more likely to suffer from morbidities such as decreases in cognitive performance [3,4], as well as an increased C-reactive protein level due to systemic inflammation [5]. Moreover, an AHI ≥5 e/h is also associated with increased systemic blood pressure measurements and an increased risk for cardiac strain [3]. All these important negative consequences highlight the necessity of an early detection of moderate-to-severe pediatric SAHS [3].

Notwithstanding the highly promising results of our current approach, several limitations must be considered. First, the exclusive use of the SpO_2_ signal to detect SAHS may restrict the spectrum of physiological perturbations being detected by the oximetry signal, such as electroencephalographic arousals or reductions in airflow and increased intrathoracic pressure swings [1]. In this regard, the combination of SpO_2_ with other physiological signals from PSG could potentially enhance the performance of our proposed method but at the cost of adding significant complexity to the test. In addition, future research efforts may prospectively focus on identifying a specific mother wavelet for this task. However, our proposed approach achieved high performance with the Haar’s mother wavelet. Of note, the lack of universally accepted AHI severity cutoffs is another limitation that affects our study. Nevertheless, we have assessed the diagnostic ability of our proposal using an AHI cutoff of 5 e/h, a widely used criterion in the clinical decision making leading to the recommendation of surgical treatment [3,10]. Finally, it would be an interesting future goal to further validate our methodology in a larger sample of unattended oximetry recordings obtained at patients’ homes.

## Conclusions

The application of WT has enabled the identification of features with the ability to characterize the effects of SAHS in the overnight oximetry profile of children. Features computed in the *D*_9_ detail level of the DWT as well as *WE* reached significant differences associated with the presence of SAHS. DWT has been found to provide complementary information to conventional approaches. Additionally, high diagnostic performance was reached using different reference binary classifiers, which emphasizes the usefulness of the DWT to provide discriminant information from oximetry signals. These results suggest that wavelet analysis could be useful to further characterize the oximetry signal and improve the diagnostic performance and implementation of abbreviated screening test for pediatric SAHS.

## Supporting information

S1 Table Actual AHI from PSG and values of all the extracted features from the SpO_2_ signal (*ODI*3, statistical moments, PSD, and DWT features) of each subject in the optimization set. (XLSX)

S2 Table The number of times each feature was selected with FCBF in the optimization set. (XLSX)

S3 Table Actual AHI from PSG, values of all the extracted features from the SpO_2_ signal (*ODI*3, statistical moments, PSD, and DWT features), and the classification scores of LR, SVM, and MLP of each subject in the cross-validation set. (XLSX)
